# Self-perceived competence in managing obstetric emergencies among recently graduated physicians from Lima, Peru

**DOI:** 10.1186/s12909-023-04854-5

**Published:** 2023-11-16

**Authors:** Wendy Nieto-Gutierrez, Alvaro Taype-Rondan

**Affiliations:** 1 Universidad Científica del Sur, Lima, Peru; 2 Universidad San Ignacio de Loyola, Unidad de Investigación para la Generación y Síntesis de Evidencias en Salud, Lima, Peru; 3 EviSalud – Evidencias en Salud, Lima, Peru

**Keywords:** Education, Medical, Education, Medical, Undergraduate, Internship, Nonmedical, Pregnancy Complications (source: MeSH)

## Abstract

**Objective:**

This study aimed to examine the self-perception of competencies in obstetric emergencies among recently graduated physicians from universities in Lima, Peru; and to identify its associated factors.

**Methods:**

An analytical study was conducted, with the study population comprising newly graduated doctors who attended the “VI SERUMS National Convention” in 2017. We used Poisson regressions to assess the factors associated with the self-perception of competencies in obstetric emergencies, calculating prevalence ratios (PR) and their 95% confidence intervals (95% CI).

**Results:**

We analyzed a population of 463 newly graduated physicians (mean age: 25.9 years), of which 33.3% reported feeling competent in obstetric emergencies. In the adjusted analyses, we found that having a previous health career (PR: 1.77, 95% CI: 1.12—2.81), having completed the internship in EsSalud hospitals (PR: 1.48, 95% CI: 1.31—1.68), and completing a university externship (PR: 1.62, 95% CI: 1.34—1.96) were associated with a higher prevalence of self-perceived competence in obstetric emergencies.

**Conclusion:**

Our findings suggest that certain academic factors, such as completing an externship and internship in specific hospital settings, may enhance the competencies or competence self-perception of recently graduated physicians in obstetric emergencies. Further studies are needed to confirm these results and identify other factors that may impact physicians’ competencies in this field.

**Supplementary Information:**

The online version contains supplementary material available at 10.1186/s12909-023-04854-5.

## Introduction

The medical education landscape has undergone significant changes in recent years [1], with a shift towards competency-based learning [2, 3], which could oscillate depending on the necessity of each country. In underdeveloped countries such as Peru, there is a pressing need to develop a broader range of skills for general physicians, particularly in the area of obstetrics, due to a shortage of multidisciplinary teams, especially in rural areas [4], where the physician can be the only healthcare professional available to provide emergency care [5], with obstetric emergencies being a frequent occurrence [6].

Much like in other Latin American countries, in Peru, obstetric emergencies are a prominent contributor to both mortality and morbidity rates, with primary care serving as the initial point of reference for these cases [7], particularly in rural areas [8]. These emergencies are estimated to contribute to nearly 25% of deaths in the referenced cases, and this percentage could potentially be even higher if the initial management is not executed with precision [9]. Given this scenario, it is imperative that primary care general physicians (that most of them are recently graduated), possess the necessary skills for the primary management and support of obstetric emergencies [6]. Despite this, reports suggest that they are not adequately equipped to effectively address such critical situations [5, 10], which has become an additional gap to an already deficient health system.

Thus, the objective of this study was to describe the self-perceived competence in managing obstetric emergencies among recently graduated physicians from Lima, Peru; and evaluate its associated factors.

## Methods

### Study design and population

In this cross-sectional study, we performed a secondary analysis of the previously collected database that aimed to assess the self-perceived competence of medical competencies, as published elsewhere [11].

The primary study encompassed a population of physicians who participated in the “VI National SERUMS Convention” organized by the “Colegio Médico del Perú,” held in Lima in April 2017. This convention aimed to gather all physicians who had recently graduated from medical schools in Lima and were preparing to commence the “Servicio Rural Urbano Marginal” (SERUMS), a mandatory healthcare service for newly graduated physicians in rural and underserved areas of Peru, with a duration of 12 months [12].

Out of the 693 physicians who registered for the SERUMS convention, 520 (75%) were included in the primary study, which represents approximately 14% of all physicians who started SERUMS in 2017 [5].

This secondary analysis only considered physicians who had completed their undergraduate studies at a university located in Lima, Peru, finished their gynecology-obstetrics internship at a hospital in Lima, Peru, completed their medical internship in 2016, and had a complete data for the outcome variable.

### Questionary

The data collection for the primary study was done using a self-administered structured questionnaire that was validated by expert judgment from methodologists and gynecologist experts, as well as through a pilot study with five newly graduated physicians who did not attend the convention. The questionnaire consisted of six sections: 1) sociodemographic data of the participant and hospital sites, 2) perception of the medical internship, 3) competencies in general medicine, 4) competencies in obstetric care, 5) competencies in gynecology, and 6) skills in mental health. In this secondary analysis, only sections 1, 2, and 4 were used (Supplementary 1).

### Procedures

Before administering the questionnaire, a succinct study overview and procedural guideline for the questionnaire were presented at the outset of the event, ensuring participants’ accurate engagement with the survey. Participants were given opportunities to complete the survey before the event’s start and during breaks. In order to address any participant queries, two researchers were available throughout the survey administration. The data underwent a dual-entry process conducted by separate researchers to ensure accuracy. An additional researcher, not previously involved in this stage, conducted a cross-validation of the double-entered data. In instances where inconsistencies were identified, a comprehensive review of the surveys was conducted to rectify any errors.

### Outcome: self-perception of obstetric emergency competence

The outcome was evaluated through the obstetric competence section of the questionnaire. The obstetric emergencies included were preeclampsia, postpartum hemorrhage, and sepsis; which were considered the most frequent obstetric emergencies [13] and the most critical for a physician’s successful performance in their SERUMS work environment [6].

For each obstetric emergency, the questionnaire included questions about the basic procedures for managing the emergency at the primary level of health care. These procedures were based on minimum competencies and standards for accreditation of medical schools in Peru as proposed by the “Comisión para la Acreditación de Facultades o Escuelas de Medicina Humana” (CAFME 2013) [14], the national clinical practice guideline for obstetric emergency care in Peru [15], and the core clinical competencies in obstetrics and gynecology for undergraduate students in Australia and Canada [16, 17].

The physician’s perception of their competence in managing the emergency was evaluated through the question: “I have the appropriate skills to perform these procedures during the SERUMS…”, with responses scored on a Likert scale from “strongly disagree” (1) to “strongly agree” (5).

To calculate the score for each emergency (preeclampsia, postpartum hemorrhage, and sepsis), the scores obtained for each of the basic procedures were averaged. The final average was then categorized as “non-competent” (score less than four) or “competent” (score greater than or equal to four). The “self-perception of obstetric emergency competence” was obtained by averaging the scores for each individual emergency topic (preeclampsia, postpartum hemorrhage, and sepsis) and was categorized as “not competent” (< 4 points) or “competent” (≥ 4 points).

### Other variables

Other assessed variables were sex, age, marital status, previous health career, university, satisfaction of teaching during the gynecology/obstetrics internship rotation (thought the question: Was the clinical teaching [during medical visits, academic activities, shift changes, etc.] in the Gynecology-Obstetrics rotation excellent?), institution where the internship was held, and externship.

The externship is a one-year university program completed prior to the medical internship. Its objective is to integrate the knowledge of the four basic areas of medicine (medicine, surgery, gynecology and obstetrics, and pediatrics) through hospital practices [18]. To construct this variable, we reviewed the university curricula for 2016 from each of the medical schools in Lima. Based on this information, we created a variable with two categories: those without externship and those with externship.

### Statistical analysis

We used Stata v.15 software for statistical analysis. Descriptive analysis was performed using absolute and relative frequency measures. To evaluate factors associated with self-self-perceived competencies in obstetric emergencies we used Poisson regression models, taking into account the university where participants graduated as a cluster. This enabled us to obtain prevalence ratios (PR) with their respective 95% confidence intervals (95% CI). To construct the adjusted model, we identified potential confounders by analyzing causal diagrams.

## Results

We evaluated a total of 520 physicians, of which we excluded 41 who did not complete their medical studies at a university in Lima and 16 who did not complete their internship in 2016. Finally, we analyzed 463 recently graduated physicians, of whom 55.1% were male and 36.8% were aged between 25 and 26 years old, and 73.0% completed their medical studies at private universities. During their internship, 67.4% of the physicians worked in a hospital under the Ministry of Health in Peru, and 67.4% reported being satisfied with the teaching during their obstetrics and gynecology rotation. Only 20.48% of the included physicians completed the externship. Although 33.3% of the physician perceived themselves competent in the management of obstetric emergencies, when we evaluated for individual emergencies, 64.1%, 54.1%, and 35.0% of the physician perceived themselves competent with the management of sepsis, postpartum hemorrhage, and preeclampsia, respectively Table 1.

**Table 1:** Characteristics of the study population (*n* = 463)

Characteristics	n (%)
**Sex**	
Female	206 (44.9)
Male	253 (55.1)
**Age in years**	25.9 ± 2.7^a^
22 to 24	149 (33.4)
25 to 26	164 (36.8)
27 to 42	133 (29.8)
**Marital status: single**	433 (94.8)
**Previous health career**	16 (3.5)
**University funding: private**	335 (73.0)
**Institution of the internship hospital**	
Ministry of Health (MINSA)	306 (67.4)
Social Health Insurance (EsSalud)	71 (15.6)
Other (private clinics, etc.)	77 (17.0)
**Satisfied with the teaching during the gynecology/obstetrics internship rotation**	308 (67.4)
**Externship**	94 (20.5)
**Self-perceived as competent in:**	
Obstetric emergency competences (average)	143 (33.3)
Preeclampsia management competences	242 (54.1)
Postpartum hemorrhage management competences	153 (35.0)
Sepsis management competences	289 (64.1)

^a^Mean ± Standard deviation

In the crude model, we observed that the prevalence of self-perceived competence in managing obstetric emergencies was higher in the group that completed an externship (PR: 1.43; 95% CI: 1.15–1.79). This finding was also observed in the adjusted model (PR: 1.62; 95% CI: 1.34–1.96). Furthermore, in the adjusted model, we found significant associations between self-perception of obstetric emergency competence and having completed a previous career in health (PR: 1.77; 95% CI: 1.12–2.81), as well as having completed the gynecology and obstetrics internship rotation in EsSalud (PR: 1.48; 95% CI: 1.31–1.68) Table 2.

**Table 2:** Factors associated with the self-perception competence in managing obstetric emergencies (*n* = 463)

Characteristics	Self-perception of obstetric emergency competences		Crude PR (95% CI)	Adjusted PR * (95% CI)
	No competent; *n* (%)	Competent; *n* (%)		
**Sex**				
Female	127 (66.8)	63 (33.2)	Ref	Ref
Male	159 (66.5)	80 (33.5)	1.01 (0.79—1.29)	1.02 (0.77—1.34)
**Age in years**				
22 to 24	91 (64.5)	50 (35.5)	Ref	Ref
25 to 26	111 (71.6)	44 (28.4)	0.80 (0.59—1.08)	0.75 (0.55—1.02)
27 to 42	80 (64.0)	45 (36.0)	1.02 (0.70—1.48)	1.01 (0.67—1.50)
**Previous health career**				
No	277 (67.2)	135 (32.8)	Ref	Ref
Yes	6 (46.2)	7 (53.8)	**1.64 (1.12 -2.40)**	**1.77 (1.12—2.81)**
**Institution of the internship hospital**				
Ministry of Health (MINSA)	199 (68.2)	93 (31.8)	Ref	Ref
Social Health Insurance (EsSalud)	37 (56.9)	28 (43.1)	**1.35 (1.08—1.68)**	**1.48 (1.31—1.68)**
Other (private clinics, etc.)	49 (72.1)	19 (27.9)	0.87 (0.58—1.31)	0.89 (0.62—1.28)
**Satisfaction with the teaching during the gynecology/obstetrics internship rotation**				
Not satisfied	98 (71.0)	40 (29.0)	Ref	Ref
Satisfied	188 (64.6)	103 (35.4)	1.23 (0.84—1.79)	1.31 (0.89—1.91)
**Externship**				
No	236 (69.4)	104 (30.6)	Ref	Ref
Yes	50 (56.2)	39 (43.8)	**1.43 (1.15—1.79)**	**1.62 (1.34—1.96)**

The highlighted values correspond to statistically significant values (*p* < 0.05)

*Ref* Reference, *PR* Prevalence ratio, *95%CI* Confidence interval at 95%

^*^ Adjusted for age, sex, previous career, location of the medical internship, externship, and rotating teaching of obstetrics and gynecology

^*^ *p*-value obtained from the chi-square test

When we evaluated each of the obstetric emergencies, we found that for physicians who completed externships, the prevalence of self-perceived competence in managing preeclampsia (PR: 1.05; 95% CI: 1.01–1.08) was higher compared to the group that did not complete an externship. This finding was consistent in the adjusted model (OR: 1.30; 95% CI: 1.28–1.32). Similarly, we observed a statistically significant association between completing an externship and self-perceived competence in managing sepsis, but only in the adjusted model (OR: 1.17; 95% CI: 1.03–1.33). However, we did not find a consistent significant association between completing an externship and self-perceived competence in managing postpartum hemorrhage Table 3.

**Table 3:** Factors associated with the self-perception competence in managing preeclampsia, postpartum hemorrhage, and sepsis (*n* = 463)

Characteristics	Preeclampsia		Postpartum hemorrhage		Sepsis	
	**Crude PR (95% CI)**	**Adjusted PR**^a^** (95% CI)**	**Crude PR (95% CI)**	**Adjusted PR**^a^** (95% CI)**	**Crude PR (95% CI)**	**Adjusted PR**^a^** (95% CI)**
**Sex**						
Female	Ref	Ref	Ref	Ref	Ref	Ref
Male	0.95 (0.78—1.17)	0.96 (0.77—1.20)	1.00 (0.80—1.25)	1.01 (0.79—1.30)	1.04 (0.90—1.20)	1.05 (0.90—1.23)
**Age in years**						
22 to 24	Ref	Ref	Ref	Ref	Ref	Ref
25 to 26	0.93 (0.78—1.11)	0.90 (0.76—1.06)	0.98 (0.78—1.23)	0.97 (0.73—1.29)	0.90 (0.79—1.02)	**0.89 (0.80—0.99)**
27 to 42	1.01 (0.80—1.28)	0.97 (0.75—1.25)	1.23 (0.83—1.83)	1.21 (0.83—1.77)	**0.82 (0.68—0.98)**	**0.82 (0.71—0.95)**
**Previous health career**						
No	Ref	Ref	Ref	Ref	Ref	Ref
Yes	**1.50 (1.12—2.00)**	**1.70 (1.19—2.44)**	**1.87 (1.26—2.77)**	1.63 (1.00—2.65)	**1.27 (1.03—1.56)**	**1.42 (1.04—1.94)**
**Institution of the internship hospital**						
Ministry of Health (MINSA)	Ref	Ref	Ref	Ref	Ref	Ref
Social Health Insurance (EsSalud)	1.15 (0.84—1.57)	1.24 (0.93—1.66)	1.22 (0.88—1.69)	1.30 (1.00—1.69)	1.17 (0.98—1.39)	1.23 (1.05—1.43)
Other (private clinics, etc.)	0.82 (0.65—1.02)	0.87 (0.72—1.05)	0.93 (0.65—1.34)	0.99 (0.69—1.44)	0.80 (0.69—0.92)	0.85 (0.72—1.01)
**Satisfaction with the teaching during the gynecology/obstetrics internship rotation**						
Not satisfied	Ref	Ref	Ref	Ref	Ref	Ref
Satisfied	**1.42 (1.16—1.74)**	**1.53 (1.25—1.87)**	1.05 (0.79—1.39)	1.08 (0.81—1.42)	**1.22 (1.06—1.41)**	**1.27 (1.08—1.49)**
**Externship**						
No	Ref	Ref	Ref	Ref	Ref	Ref
Yes	**1.05 (1.03—1.07)**	**1.30 (1.11—1.53)**	0.94 (0.73—1.20)	1.00 (0.78—1.29)	1.11 (0.94—1.32)	**1.17 (1.03—1.33)**

The highlighted values correspond to statistically significant values (*p* < 0.05)

*Ref* Reference, *PR* Prevalence ratio, *95%CI* Confidence interval at 95%

^a^Adjusted for age, sex, previous career, location of the medical internship, rotating teaching of obstetrics and gynecology

## Discussion

### Competencies in obstetric emergencies

In Peru, the majority of recently graduated physicians begin their careers by participating in SERUMS at the primary level of care [5, 19] in regions characterized by high vulnerability and a shortage of multidisciplinary staff [5]. As a result, these physicians need to possess the necessary knowledge and competencies to effectively manage prevalent conditions, including obstetric emergencies [8].

Our study identified that approximately one-third of recent medical graduates perceived themselves as lacking competence in managing obstetric emergencies. This finding is consistent with a study conducted in Chile, where 60% of physicians self-perceived as a non-competent in obstetric management [20]. The high percentage of self-perception is likely due to shortcomings in medical education in Peru, where there is a disconnect between the primary care competencies taught in universities and the competencies needed for the workforce as postulated by the Ministry of Health [19]. Furthermore, it is crucial to acknowledge that medical education has predominantly focused on inpatient settings, despite the fact that the practice of medicine primarily occurs in outpatient settings [21], especially at the beginning of the work career. This circumstance, compounded by restricted resources and logistical hurdles, results in physicians gaining their initial clinical experience in the most intricate and fast-paced healthcare environments, which presents a heightened level of challenge [21].

In Peru, the essential training for emergency management competence also primarily occurs within hospital settings [22]. This educational approach might have been impacted by the proliferation of medical colleges and the subsequent rise in number of students [23]. Consequently, a heightened concentration of students within healthcare institutions leads to a shortage of clinical settings for procedural training, a deficiency in available instructors, and constrained instructional hours [24]. This is a concerning situation, as practical healthcare training typically necessitates a maximum of one student per patient in emergency care scenarios [25]. With the notable growth of medical colleges, particularly in Lima, over the past decade [23] coupled with the substantial number of medical students present in clinical areas [26], there is a possibility that this established standard is not being upheld.

When we evaluated self-perceived competencies for each topic, we found that more than half of the participants perceived themselves as non-competent in postpartum hemorrhage management. This outcome is likely attributed to the fact that effective management of postpartum hemorrhage entails both pharmacological intervention and the accurate execution of maneuvers for placental removal [27]. These aspects can present increased challenges and demand more comprehensive training. Furthermore, given that postpartum hemorrhage demands prompt intervention due to its potential for rapid hemodynamic instability and maternal mortality [28]; certain healthcare facilities might not afford students the chance to manage this type of condition, consequently leading to a deficit in practical experience.

International experiences suggest that for the development of emergency competencies, students need clinical training with real cases supervised by experienced instructors [22]; Nevertheless, it’s equally vital to integrate simulation exercises, including hands-on practice of procedures like placental removal using models, virtual programs, and other educational tool [22, 29], to increase the clinical practice and minimize the risk of poor results in obstetric emergency management. Inclusive, this teaching class can also be offered as an elective course at the beginning of the medical career, allowing students the opportunity to enroll in a quarter-long course focused on teaching and practicing common obstetric emergency procedures in the early stages of their careers, as reported in a previous study [30].

### Associated factors

A higher proportion of physicians that self-perceived competent in general emergencies have done a university externships. Probably it is because the externship inserts the medical students in a clinical environment, entailing them to get competencies in real practice and direct observation [31]. This was corroborated by previous studies that reported results about the favorable impact of direct observation in the formation of procedural competencies [32] with good acceptance from students and teachers [33, 34].

Although our study found a significant association between completing a university externship and self-perceived competencies in managing obstetric emergencies, it’s important to note that in the sub-analysis examining individual pathologies, this association was not observed in the context of postpartum hemorrhage management. This is probably because postpartum hemorrhage involves complex management [28], making it challenging to attain an optimal self-perception level for this competence even with a university externship. This result also suggests that obstetric competencies should be assessed individually, encouraging tailored learning strategies for each topic based on the disease’s characteristics and its management [35].

To ensure effective learning during university externships or internships, it’s essential to take into account the type of institution in which they are conducted, as each institution provides distinct clinical opportunities. This can result in an imbalance of the knowledge acquired between institutions. For instance, students who participate in externships at specialized obstetric institutions are likely to receive more focused teaching on this topic, with greater opportunities for obstetric procedural learning. This has been reported in studies conducted in the United States [22] and Iran [36], which recommend prioritizing clinical practices in specialized rooms or centers to enhance opportunities for patient attention and emergency procedures. Therefore, it is crucial to link externship implementation to continuous monitoring by the university, establishing indicators to evaluate procedural learning in each student (number of processes completed in each topic by each student) and designating a specified time for learning each basic competency in obstetrics.

Our study found that physicians who had previously completed a career in health had a higher prevalence of perceiving themselves as competent. This is not surprising as self-perception of competencies is closely linked to confidence [37] and therefore professionals with prior knowledge and experience would likely have greater confidence in managing diseases and subsequently a better self-perception of their abilities.

Additionally, our study revealed that the type of institution where the medical internship was completed was associated with self-perception of competencies. Previous studies conducted in Peru [38] and Brazil [39] have also reported the fundamental role of internships in the acquisition of skills in obstetrics and gynecology. However, our study only found a higher prevalence of physicians who perceived themselves as competent when they completed their medical internship at an EsSalud hospital. In Peru, internship institutions display notable disparities in logistic resources (such as teaching resources), clinical areas, and patient demand [40]. Our results are as expected, given the renowned reputation of EsSalud hospitals for their exceptional teaching performance, especially in the procedural aspects of obstetrics and gynecology [41]. Additionally, in Peru, gaining an internship placement at an EsSalud institution necessitates passing a knowledge examination and maintaining acceptable performance during undergraduate studies [42]. This requirement may contribute to students having a stronger knowledge base and, consequently, a heightened self-perception of their competencies.

It is important to note that self-perception of competencies may not necessarily reflect actual competencies, and therefore continuous monitoring and evaluation of skills acquisition through objective measures is crucial for ensuring quality medical education.

### Limitations and strengths

Some limitations of our study must be taken into account. First, the sample was gathered through a non-probabilistic method, encompassing physicians who participated in a Peruvian national convention. This introduces a selection bias, potentially leading to a greater likelihood of including participants with a more positive self-perception and, consequently, an overestimation of the results. Nonetheless, our findings align consistently with prior studies conducted in comparable contexts in other countries [20, 43–45] bolstering the certainty of the evidence.

Additionally, our evaluation of obstetric competencies relied on self-perception rather than direct assessment of clinical skills. While self-perception has shown to be a useful measure of self-efficacy, it may not necessarily reflect actual competence levels [46], which could potentially affect our results [37]. However, we believe that our findings remain informative, as self-perception is linked to both real knowledge and the interest to learn and improve one’s skills [47, 48].

On the other hand, our study is among the first in the region to examine self-perceived competencies among physicians managing obstetric emergencies, providing important insights into the skills and knowledge levels of physicians in this field. Our findings highlight the need for interventions to improve obstetric emergency management, particularly given the high mortality rate associated with this condition in our context.

## Conclusion

Our study found that having a previous health career, having performed the internship in EsSalud hospitals, and completing a university externship, were associated with a higher prevalence of self-perceived competence in obstetric emergencies among recently graduated physicians. These findings suggest that certain academic factors during the last years of study can be critical to improve the competencies or competence self-perception in physicians. Further studies are needed to confirm these results and identify other factors that may impact physicians’ competencies in this field.

### Supplementary Information

**Additional file 1.**

## Data Availability

https://figshare.com/articles/dataset/Database_BMC/22280695.
