# Supplementary material for: Self-perceived competence in managing obstetric emergencies among recently graduated physicians from Lima, Peru
Source: BMC Med Educ. 2023 Nov 16;23:876. doi: 10.1186/s12909-023-04854-5 (PMC10655440; doi:10.1186/s12909-023-04854-5)
Supplement: Supplementary file 1 — Additional file 1. [file 12909_2023_4854_MOESM1_ESM.docx]

# SUPPLEMENTARY MATERIAL

## Supplementary 1. Questionary for evaluating self-perception in managing obstetric emergencies

**Indication:** Please mark with an "x" [X] or fill in the blanks ___________ with clear handwriting, as appropriate.

| **SECTION 1. Sociodemographic variables**    1. Age (in years): ________________  2. Sex:  1 [ ] Male  2 [ ] Female  3. Current marital status:  1 [ ] Single  2 [ ] Married  3 [ ] Other_____________  4. Did you complete any other health-related bachelor before studying medicine? Please specify which one:  0 [ ] No  1 [ ] Yes, midwifery  2 [ ] Yes, nursing  3 [ ] Yes, psychology  4 [ ] Yes, other (Please specify which one): ________________  5. Undergraduate university where you studied:  1 [ ] Nacional Mayor de San Marcos university  2 [ ] Peruana Cayetano Heredia university  3 [ ] Peruana de Ciencias Aplicadas university  4 [ ] Nacional Federico Villareal university  5 [ ] San Martín de Porres university  6 [ ] Ricardo Palma university  7 [ ] Privada San Juan Bautista university  8 [ ] Científica del Sur university  9 [ ] Nacional José Faustino Sánchez Carrión university | 6. In which city did you study your undergraduate bachelor in medicine?  1 [ ] Lima  2 [ ] Other: _______________________  7. Have you taken part in any externships at any time during the last 3 years of your bachelor's degree? (Extracurricularly)  1 [ ] No  2 [ ] Yes, but less than 2 months  3 [ ] Yes, 2 months or more  **SECTION 2. Internship**  8. In which year did you complete your internship?  1 [ ] 2016  2 [ ] 2015  3 [ ] 2014  4 [ ] Before of 2014  9. Where did you complete your internship in medicine bachelor? (You can select more than one option, if applicable)  1 [ ] “MINSA”  2 [ ] “EsSalud”  3 [ ] “Sanidades”  4 [ ] Private clinic |
| --- | --- |

**SECTION 3. Perception of Medical Internship:** Please indicate your level of agreement with the following statements about your internship experience by marking with an "x".

|  | **1**  **Strongly Disagree** | **2**  **Disagree** | **3**  **Neutral** | **4**  **Agree** | **5**  **Strongly Agree** |
| --- | --- | --- | --- | --- | --- |
| Did **paperwork** (filling out forms, processing plans, etc.) occupy most of your time… |  |  |  |  |  |
| 1. ...during the Internal Medicine rotation? |  |  |  |  |  |
| 1. ...during the General Surgery rotation? |  |  |  |  |  |
| 1. ...during the Gynecology rotation? |  |  |  |  |  |
| 1. ...during the Pediatrics rotation? |  |  |  |  |  |
| Was the **clinical instruction** (during medical rounds, academic activities, shift changes, etc.) provided excellent... |  |  |  |  |  |
| 1. ...during the Internal Medicine rotation? |  |  |  |  |  |
| 1. ...during the General Surgery rotation? |  |  |  |  |  |
| 1. ...during the Gynecology rotation? |  |  |  |  |  |
| 1. ...during the Pediatrics rotation? |  |  |  |  |  |

**SECTION 4. Obstetric Care Competencies:** Please mark with an "x" as appropriate:

| ***I have the necessary competencies to carry out these procedures during the SERUMS…*** | **1**  **Strongly Disagree** | **2**  **Disagree** | **3**  **Neutral** | **4**  **Agree** | **5**  **Strongly Agree** |
| --- | --- | --- | --- | --- | --- |
| **In case of Preeclampsia:** |  |  |  |  |  |
| Administer magnesium sulfate in cases of severe preeclampsia |  |  |  |  |  |
| Insert foley catheter and monitor hourly diuresis |  |  |  |  |  |
| Administer nifedipine in cases of severe preeclampsia |  |  |  |  |  |
| **In case of postpartum hemorrhage because of uterine atony** |  |  |  |  |  |
| Administer intravenous oxytocin |  |  |  |  |  |
| Perform bimanual uterine massage in cases of uterine atony (massage the uterus downward from the abdomen with one hand and upward from the vagina with the other hand) |  |  |  |  |  |
| Insert a second intravenous line with only NaCl, infuse 500cc rapidly, and then continue at a rate of 30 drops per minute |  |  |  |  |  |
| Perform manual extraction of placental remnants in cases of postpartum hemorrhage caused by retained placental fragments |  |  |  |  |  |
| Administer intramuscular ergometrine in cases of uterine atony |  |  |  |  |  |
| **In case of obstetric sepsis** |  |  |  |  |  |
| Administer intravenous ampicillin and gentamicin in cases of puerperal sepsis |  |  |  |  |  |
| Detect signs of Systemic Inflammatory Response Syndrome (SIRS) |  |  |  |  |  |

## Supplementary 2. Association between self-perception of obstetric emergency competence (score) and externship in regression analysis, epidemiological approach

| **Characteristics** | | **Preeclampsia** | | **Postpartum hemorrhage** | | **Sepsis** | | **Emergency competence** | |
| --- | --- | --- | --- | --- | --- | --- | --- | --- | --- |
|  |  | **Crude PR (95% CI)** | **Adjusted PR***  **(95% CI)** | **Crude PR (95% CI)** | **Adjusted PR* (95% CI)** | **Crude PR (95% CI)** | **Adjusted PR***  **(95% CI)** | **Crude PR (95% CI)** | **Adjusted PR* (95% CI)** |
| Gender | | | | | | | | | |
|  | Female | Ref. | Ref. | Ref. | Ref. | Ref. | Ref. | Ref. | Ref. |
|  | Male | -0.03  (-0.19 - 0.13) | -0.03  (-0.22 - 0.17) | 0.02  (-0.10 - 0.15) | 0.02  (-0.13 - 0.16) | 0.06  (-0.07 - 0.19) | 0.06  (-0.10 - 0.21) | 0.03  (-0.10 - 0.16) | 0.02  (-0.13 - 0.18) |
| Age | |  |  |  |  |  |  |  |  |
|  | 22 and 24 years old | Ref. | Ref. | Ref. | Ref. | Ref. | Ref. | Ref. | Ref. |
|  | 25 and 26 years old | -0.05  (-0.25 - 0.15) | -0.07  (-0.27 - 0.12) | -0.04  (-0.20 - 0.11) | -0.07  (-0.23 - 0.10) | -0.11  (-0.32 - 0.10) | -0.14  (-0.34 - 0.07) | -0.07  (-0.26 - 0.11) | -0.10  (-0.28 - 0.08) |
|  | 27 and 42 years old | -0.05  (-0.21 - 0.11) | -0.08  (-0.27 - 0.11) | 0.05  (-0.15 - 0.26) | 0.03  (-0.16 - 0.22) | -0.11  (-0.26 - 0.05) | -0.11  (-0.26 - 0.04) | -0.03  (-0.20 - 0.14) | -0.06  (-0.23 - 0.11) |
| **Previous health careers** | | | | | | | | | |
|  | No | Ref. | Ref. | Ref. | Ref. | Ref. | Ref. | Ref. | Ref. |
|  | Yes | 0.29  (0.00 - 0.59) | **0.40**  **(0.09 - 0.71)** | **0.55**  **(0.11 - 0.99)** | **0.56**  **(0.08 - 1.04)** | 0.29  (-0.09 - 0.67) | **0.36**  **(0.02 - 0.71)** | **0.44**  **(0.10 - 0.77)** | **0.50**  **(0.13 - 0.87)** |
| **Institution of the internship hospital** | | | | | | | | | |
|  | Ministry of Health (MINSA) | Ref. | Ref. | Ref. | Ref. | Ref. | Ref. | Ref. | Ref. |
|  | Social Health Insurance (EsSalud) | 0.11  (-0.06 - 0.28) | **0.17**  **(0.03 - 0.30)** | 0.11  (-0.04 - 0.26) | 0.16  (0.00 - 0.32) | 0.18  (0.08 - 0.28) | 0.21  (0.13 - 0.29) | **0.13**  **(0.06 - 0.20)** | **0.18**  **(0.10 - 0.26)** |
|  | Other (private clinics, etc.) | -0.20  (-0.40 - 0.00) | -0.18  **(-0.31 - -0.05)** | -0.14  (-0.30 - 0.02) | -0.12  (-0.27 - 0.04) | -0.09  (-0.27 - 0.09) | -0.06  (-0.25 - 0.13) | **-0.13**  **(-0.30 - 0.05)** | -0.10  (-0.25 - 0.05) |
| **Satisfaction about teaching in the gynecology and obstetrics internship rotation** | | | | | | | | | |
|  | Not satisfied | Ref. | Ref. | Ref. | Ref. | Ref. | Ref. | Ref. | Ref. |
|  | Satisfied | **0.18**  **(0.05 - 0.32)** | **0.20**  **(0.07 - 0.34)** | 0.10  (-0.05 - 0.24) | 0.11  (-0.04 - 0.26) | **0.17**  **(0.03 - 0.32)** | **0.20**  **(0.03 - 0.36)** | **0.15**  **(0.03 - 0.26)** | **0.17**  **(0.04 - 0.29)** |
| **Externship** | | | | | | | | | |
|  | No | Ref. | Ref. | Ref. | Ref. | Ref. | Ref. | Ref. | Ref. |
|  | Yes | **0.18**  **(0.09 - 0.26)** | **0.24**  **(0.16 - 0.32)** | 0.06  (-0.09 - 0.20) | 0.11  (-0.01 - 0.23) | **0.14**  **(0.02 - 0.25)** | **0.17**  **(0.07 - 0.28)** | **0.14**  **(0.04 - 0.24)** | **0.19**  **(0.09 - 0.29)** |

*The highlighted values correspond to statistically significant values (p<0.05)*

*Ref: reference. PR: Prevalence ratio. 95%CI: Confidence interval at 95%*

**Adjusted for age, sex, previous career, location of the medical internship, rotating teaching of obstetrics and gynecology*

## Supplementary 3. Characteristics of the gynecology and obstetrics rotation during the university externship, according to university

| **UCSUR** | **UPC** | **UPCH** |
| --- | --- | --- |
| Hospital where it is possible to do the externship (Institution to which it belongs) | | |
| - Hospital Nacional Arzobispo Loayza (MINSA) - Hospital María Auxiliadora (MINSA) - Centro Materno Infantil Juan Pablo II (MINSA) | - Hospital Edgardo Rebagliati Martins (EsSalud) - Hospital EsSalud Suárez Angamos (EsSalud) - Hospital Nacional Docente Madre Niño San Bartolomé (MINSA) - Hospital EsSalud Vitarte (EsSalud) | - Hospital Cayetano Heredia (MINSA) - Hospital Nacional Arzobispo Loayza (MINSA) |
| Duration of the academic rotation | | |
| 3 month | 1 month | 1 month and 2 weeks |
| Were theoretical classes taught during the rotation? | | |
| Yes, in the university | No | No |
| Was there monitoring of externship rotation? | | |
| Yes, reporting the number of procedures performed in basic skills | Yes, supervising the activities by the tutor at the venue, for at least 2 hours a day. | Yes, reporting the number of procedures performed in the basic skills with a minimum number of procedures to be performed |
| Was a procedural assessment of basic skills carried out? | | |
| No | Yes, only evaluation of the completion of obstetric clinical history and delivery care | Yes, only delivery care evaluation and papanicolau procedure |

*UCSUR: Universidad Científica del Sur; UPC: Universidad Privada de Ciencias aplicadas; UPCH: Universidad Peruana Cayetano Heredia; MINSA: Ministry of Health; EsSalud:Social Health Insurance*
